# Focused Ultrasound Stimulation as a Neuromodulatory Tool for Parkinson’s Disease: A Scoping Review

**DOI:** 10.3390/brainsci12020289

**Published:** 2022-02-19

**Authors:** Keng Siang Lee, Benjamin Clennell, Tom G. J. Steward, Andriana Gialeli, Oscar Cordero-Llana, Daniel J. Whitcomb

**Affiliations:** 1 Bristol Medical School, Faculty of Health Sciences, University of Bristol, Bristol BS8 1TH, UK; mrkengsianglee@gmail.com (K.S.L.); ben.clennell@bristol.ac.uk (B.C.); ts7764@bristol.ac.uk (T.G.J.S.); ag15595@bristol.ac.uk (A.G.); oscar.corderollana@bristol.ac.uk (O.C.-L.); 2 Regenerative Medicine Laboratory, School of Clinical Sciences, University of Bristol, Bristol BS8 1TH, UK; 3 Institute of Clinical Neurosciences, Bristol Medical School, Translational Health Sciences, University of Bristol, Bristol BS8 1TH, UK

**Keywords:** neuroscience, neuromodulation, Parkinson’s disease, scoping review, ultrasound

## Abstract

Non-invasive focused ultrasound stimulation (FUS) is a non-ionising neuromodulatory technique that employs acoustic energy to acutely and reversibly modulate brain activity of deep-brain structures. It is currently being investigated as a potential novel treatment for Parkinson’s disease (PD). This scoping review was carried out to map available evidence pertaining to the provision of FUS as a PD neuromodulatory tool. In accordance with the Preferred Reporting Items for Systematic Reviews and Meta-Analysis Extension for Scoping Reviews, a search was applied to Ovid MEDLINE, Embase, Web of Science and Cochrane Central Register of Controlled Trials on 13 January 2022, with no limits applied. In total, 11 studies were included: 8 were from China and 1 each from Belgium, South Korea and Taiwan. All 11 studies were preclinical (6 *in vivo*, 2 *in vitro*, 2 mix of *in vivo* and *in vitro* and 1 *in silico*). The preclinical evidence indicates that FUS is safe and has beneficial neuromodulatory effects on motor behaviour in PD. FUS appears to have a therapeutic role in influencing the disease processes of PD, and therefore holds great promise as an attractive and powerful neuromodulatory tool for PD. Though these initial studies are encouraging, further study to understand the underlying cellular and molecular mechanisms is required before FUS can be routinely used in PD.

## 1. Introduction

Parkinson’s disease (PD) is a common and progressive neurodegenerative condition, characterised by the degeneration and death of dopaminergic neurons in the substantia nigra pars compacta (SN_pc_) and reduced dopamine biosynthesis from surviving neurons [1]. As the disease advances, PD patients can experience motor manifestations typically starting with tremor, progressing to bradykinesia and typical “cogwheel” rigidity, postural instability and gait disorders [2]. These manifestations significantly impact the activities of daily living and health-related quality of life of these patients [2,3,4]. With the global increase in life expectancy and an ageing population, both the incidence and prevalence of PD have also been rising [5,6,7], steadily becoming a major public health issue [8].

Deep brain stimulation (DBS) of either the subthalamic nucleus (STN) or internal globus pallidus (GP_i_) is a well-recognised and established neurosurgical procedure approved for advanced PD refractory to medication. Many hypotheses have been proposed for the mechanisms by which DBS generates improvements in motor symptoms, but prevailing theories have focused on stimulation-induced disruption of pathological brain circuit activity [9,10], which occur at the ionic, protein, cellular and network levels [11]. Although it is effective and in aggregate it is a safe approach [12,13,14,15], just like any surgical procedure, DBS can be a double-edged sword; it is not free of complications and may even lead to more harm to the patient [16,17,18]. A consequence of this is strict patient criteria, and thus the number of people that can actually benefit from this treatment is relatively low. Accordingly, the demand for non-invasive alternatives to open stereotactic procedures is significant.

Currently, non-invasive but irreversible neuromodulatory ablative approaches such as stereotactic radiosurgery [19], and even magnetic resonance guided high-intensity focused ultrasound (MRgFUS) for subthalatomy or pallidotomy, are clinically employed for movement disorders [20,21,22,23,24] but are not without complications.

**Figure 1:**
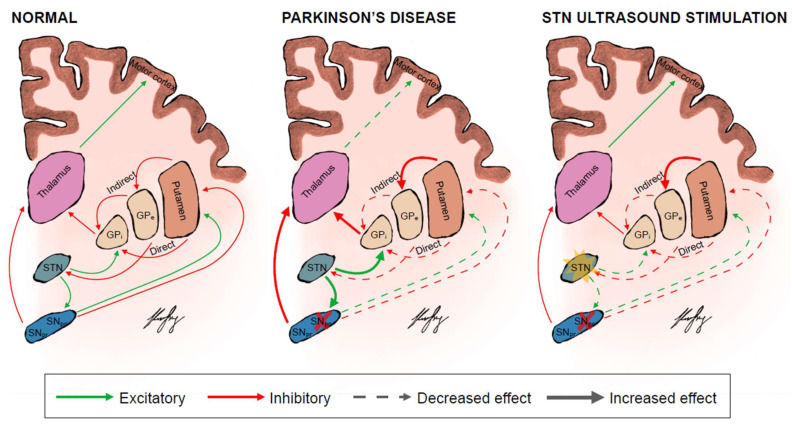
The basal ganglia circuits in a normal person, PD patient and when they are theoretically modulated by focus ultrasound stimulation of the STN. The direct pathway facilitates while the indirect pathway inhibits movement. The colours represent the excitatory (green) and inhibitory (red) neuronal pathways. The thickness and dottedness of the lines between the regions represent the strength of signalling. The red cross over the SNpc represents degeneration and death of dopaminergic neurons in the SNpc and reduced dopamine biosynthesis from surviving neurons. The yellow star over the STN represents stimulation of the target STN. GPi = globus pallidus internus; GPe = globus pallidus externus; SNpc = substanstia nigra pars compacta; SNpr = substanstia nigra pars reticulata; STN = subthalamic nucleus.

Transcranial magnetic stimulation (TMS) and transcranial current stimulation (TCS) have emerged as the cornerstone of non-invasive modulation of neural activity in particular regions of the brain [25,26,27,28]. Although promising, both TMS and TCS are limited by their broad radius of action [29]. This lack of spatial focality is particularly pronounced within the context of neuromodulating deep brain pathways such as the striato-pallido-thalamic network or deep structures including the STN and GP_i_, which are relevant to PD pathophysiology (Figure 1) [30,31]. TMS and TCS are generally constrained to targeting superficial cortical regions, as their efficacy declines exponentially with depth [30,31]. In contrast, focused ultrasound stimulation (FUS) is a non-invasive and non-ionising technique that employs acoustic energy and can acutely and reversibly modulate deep brain structures with sharp spatial resolution and non-invasive deep penetration [32,33,34]. FUS therefore holds promise as a powerful neuromodulatory tool for PD.

In order to explore the potential of FUS as a neuromodulatory tool in PD, we undertook a scoping review to profile the existing literature. To our knowledge, this is the first scoping review of its kind. The overall aims of the scoping review were to collate, map, assess and describe the existing evidence base relating to this topic, in a formal, systematic and transparent way [35]. It is intended that the findings of this review can be used to identify potential gaps in knowledge and contribute to further development of research relating to the field of FUS and PD.

## 2. Materials and Methods

This scoping review was conducted in accordance with the Preferred Reporting Items for Systematic Reviews and Meta-Analysis Extension for Scoping Reviews (PRISMA-ScR) [36]. Unlike systematic reviews, scoping reviews do not need to have a protocol registered [35]. A scoping review was chosen over a systematic review as emerging evidence relating to the provision of FUS in PD has not been comprehensively reviewed and large variation in reporting exists between studies [35,37,38].

### 2.1. Search Strategy

A search string was developed to identify original studies investigating the role of reversible low-intensity FUS (LIFUS) as a neuromodulatory tool in PD. Current clinically available ablative high-intensity FUS were beyond the scope of this review. The search terms comprised synonyms of two key concepts, namely ultrasound neuromodulation, and Parkinson’s disease. The search was applied to the following four electronic databases: Ovid MEDLINE, Embase, Web of Science and Cochrane Central Register of Controlled Trials (CENTRAL). Searches were performed for each database on 13 January 2022. No limits were applied (Supplementary Table S1).

### 2.2. Study Selection and Reliability

Articles were selected for inclusion in the review if they were published in a peer-reviewed journal in any language, and addressed the role of FUS as a neuromodulatory tool in PD. All titles and abstracts were screened independently by two reviewers (KSL and BC) against a set of pre-defined eligibility criteria. Potentially eligible studies were selected for full-text analysis. To ensure literature saturation, the reference lists of the included studies were scanned. A summary of the inclusion and exclusion criteria is presented in Supplementary Table S2. Disagreements were resolved by consensus or appeal to a third senior reviewer (DJW). Agreement among the reviewers on study inclusion was evaluated using Cohen’s kappa [39].

### 2.3. Data Extraction

A proforma was developed to conduct systematic data extraction with fields relating to (i) study design, (ii) country, (iii) subject models/participants, (iv) intervention assessed, (v) comparators against which FUS interventions are compared, (vi) data collection method and outcome measures and (vii) main findings. Two reviewers independently (KSL and BC) charted data from each eligible article. Any disagreements were resolved through discussion between the two reviewers or further adjudication by a third reviewer (DJW) until consistency was achieved.

### 2.4. Outcomes

Outcome variables were not predefined in this study, due to its exploratory rather than hypothesis-led nature.

### 2.5. Synthesis of Results

A narrative synthesis of data, with descriptive analyses where appropriate, was undertaken to enable the analysis of the relationships within and between studies, as well as assessing gaps in the literature. An analytical framework of quantitative and thematic approach was used to collate various themes that emerged from the existing data. The articles were also coded according to the categories identified in the data charting stage. Discrepancies in coding and synthesis of final frequency statistics were adjudicated by discussion.

## 3. Results

### 3.1. Characteristics of Inlcuded Studies

**Figure 2:**
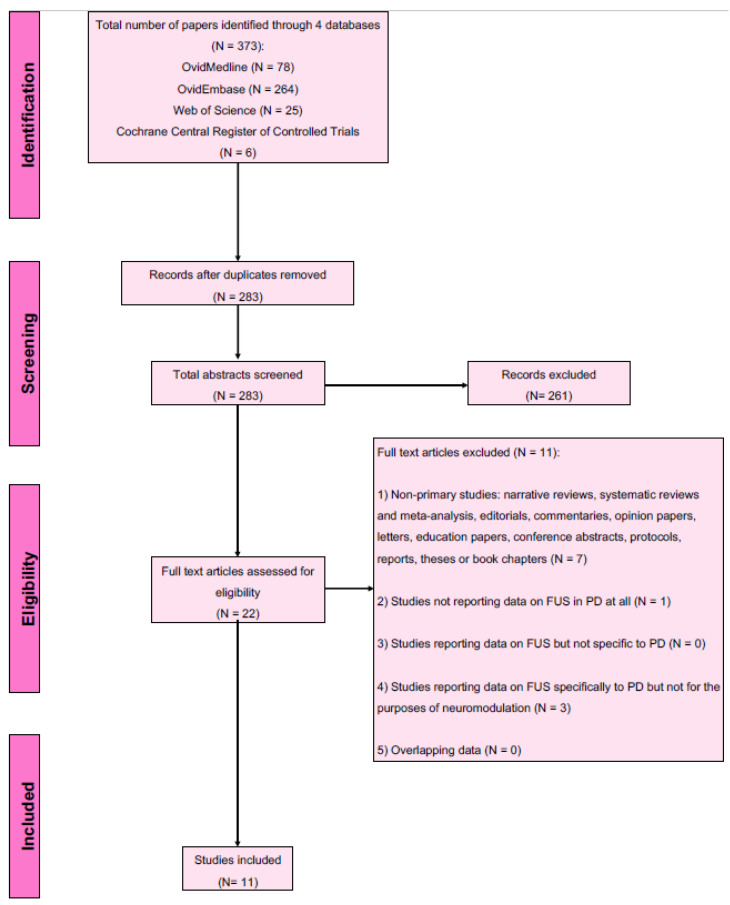
PRISMA flow diagram for studies included and excluded from the scoping review.

Number of articles screened and selected for inclusion are shown in Figure 2. Using the designated search terms, a total of 373 unique articles were identified and 11 were included in the final dataset [20,21,22,40,41,42,43,44,45,46,47,48,49,50]. Reliability of study selection between observers was substantial at both the title and abstract screening stage (Cohen’s κ = 0.94) and the full-text review stage (Cohen’s κ = 1.00) [39]. Among these 11 articles, 8 were from China and 1 each from Belgium, South Korea and Taiwan. All 11 studies were preclinical [40,41,42,43,44,45,46,47,48,49,50], including 1 computational *in silico* study [44]. The majority of the primary studies (6 of 11 (54.5%)) included were published from 2020 onwards, demonstrating the rapid pace at which the field is growing. Table 1 summarises the details and findings of all included studies.

### 3.2. Outcome Measures

An extensive range of neuromodulatory outcomes were identified (Table 1). These outcome measures were considered and categorised into two main areas: those relevant to *in vitro* models and those relevant to *in vivo* models. The most frequently investigated outcome variable employed in *in vitro* studies were improvement in cell viability/reduction in apoptosis (*n* = 3), suppression of 1-methyl-4-phenylpyridinium ion (MPP^+^)-induced reactive oxygen species (ROS) generation (*n* = 3), attenuation of MPP^+^-induced suppression of mitochondrial complex I activity (*n* = 2), suppression of MPP^+^-induced expression of casein kinase 2 (CK2) that mediates ROS-dependent α-synuclein aggregation (*n* = 1) and finally dopamine release (*n* = 1). For the *in vivo* studies, the most frequently studied outcome variable to evaluate the effectiveness of FUS was locomotor behaviour (*n* = 6) assessed using a battery of tests such as the rotarod and pole and open field forced swimming tests. The next common measures to ascertain therapeutic effects included the proportion of tyrosine hydroxylase (TH) enzyme (*n* = 5) and glial cell line-derived neurotrophic factor (GDNF)-positive neurons in the SNpc (*n* = 2). Suppression of 1-methyl-4-phenyl-1,2,3,6-tetrahydropyridine (MPTP)-induced cell apoptosis/reduction of antioxidant enzyme activity (*n* = 2) and neuronal activity using c-Fos as a surrogate was also investigated (*n* = 2). Other investigated outcomes included the reduction in iron staining in the SNpc (*n* = 1), proportion of brain-derived neurotrophic factor (BDNF)-positive neurons in the SNpc (*n* = 1), dopamine content in the SNpc (*n* = 1) and fractional anisotropy (FA) and T2* values via magnetic resonance imaging (MRI) scanning (*n* = 1) and local field potentials in the motor cortex (*n* = 1). Safety outcomes included the lack of haemorrhage and morphological changes on brain sections (*n* = 4), typically ascertained by haematoxylin and eosin (HE) and Nissl staining.

### 3.3. Trends and Findings from In Vitro Preclinical Studies

Differentiated pheochromocytoma (PC12) and N2a cells exposed to MPP^+^-induced neuronal toxicity were the most commonly studied models of PD *in vitro*. Karmacharya showed that in MPP^+^ treated PC12 cells, low-intensity ultrasound attenuated mitochondrial ROS production and reversed the inhibition of mitochondrial complex I activity by MPP^+^ [42]. When cells treated with 400 or 800 μM MPP^+^ were stimulated with 30, 50 and 100 mW/cm^2^ ultrasound for 10 min, the mitochondrial ROS production was decreased as compared to non-stimulated group as demonstrated by a reduction in the MitoSOX Red intensity, which was found to be stimulation intensity dependent [42].

Mitochondrial integrity is tightly regulated by the balance between pro-apoptotic Bax and anti-apoptotic Bcl-2 [51,52,53,54,55,56,57,58]. In the context of MPTP and MPP^+^ neurotoxic damage, Bax downregulation attenuates DA neuron apoptosis [1,53,59], whereas Bcl-2 upregulation has neuroprotective effects against the depletion of striatal dopamine [53,59,60]. Zhao and colleagues showed that low-intensity ultrasound pre-treatment increased the Bcl-2/Bax ratio, prevented Cytochrome C release and suppressed cleaved-caspase 3 activity that would all be present after MPP^+^ exposure [48]. Additionally, low-intensity ultrasound attenuated MPP^+^-induced ROS accumulation and oxidative stress in PC12 cells by modulating the expression of antioxidant proteins haem oxygenase-1 (HO-1) and thioredoxin-1 (Trx-1), and improved mitochondrial membrane potential in both PC12 cells [48] and in N2a cells [40].

Low-intensity ultrasound improved cell viability in both PC12 and N2a cells after being treated with MPP^+^ as assessed by lactate dehydrogenase (LDH), MTT and TUNEL assays [42,48]. Pre-treatment with low-intensity ultrasound suppressed the MPP+-induced increase in the level of caspase-9 cleavage products [48], suggesting that low-intensity ultrasound can modulate the mitochondrial apoptotic pathway, but not endoplasmic reticulum stress-induced apoptotic pathway. Importantly, low-intensity ultrasound decreased MPP^+^-induced α-synuclein aggregation, levels of phosphorylated α-synuclein and expression of CK2 [42], which constitutively phosphorylates α-synuclein at S129 [61,62].

These findings suggest that low-intensity ultrasound can help to maintain integrity and function of challenged mitochondria. Together, this indicates that low-intensity ultrasound inhibited MPP^+^-induced mitochondrial dysfunction and apoptosis.

### 3.4. Trends and Findings from In Vivo Preclinical Studies

With a steady increase in the number of publications relating to research of FUS in PD, contemporary animal models were most often mouse and rat species. No primate, dog or pig species were used during the study period. Induction of a PD phenotype was typically by intraperitoneal injection of MPTP in C57BL/6 mice or by intracranial injection of 6-hydroxydopamine (6-OHDA) in Sprague–Dawley rats.

In the study by Zhou et al., they observed that LIFUS (30 min daily, for 12 days) targeting either the STN or GPi, upregulated Bcl-2, downregulated Bax and inhibited increments in protein levels of both Cytochrome C and cleaved-caspase 3, thereby reversing the changes of MPTP exposure in C57BL/6 mice [49]. These findings are in agreement with the *in vitro* findings by Zhao et al. in PC12 cells [48].

TH is the rate-limiting enzyme responsible for catalysing the conversion of the amino acid L-tyrosine to L-3,4-dihydroxyphenylalanine (L-DOPA), which is eventually transformed to dopamine [63]. Multiple studies showed that LIFUS ameliorated the reduction of TH in the SNpc and striatum induced by MPTP or 6-OHDA administration as detected by immunohistochemical analyses, demonstrating the restorative effects of LIFUS on the nigrostriatal pathway [40,41,43,46]. Importantly, Xu et al. showed that this rise in TH levels was accompanied by an increase in striatal dopamine levels as measured by isocratic elution and electrochemical detection [46].

In 6-OHDA-induced Sprague–Dawley hemi-lesioned-PD rat models, LIFUS to the striatum increased the levels of GDNF in the SNpc but not in the striatum [41,43]. GDNF has potent neuroprotective and neurorestorative effects particularly, but not exclusively, on dopaminergic neurons in animal models of PD [64]. The mechanism is still unclear, but a plausible explanation for the rise in GDNF levels with low-intensity transcranial ultrasound stimulation (LITUS) could be that LITUS disrupted the BBB acutely and promoted exogenous GDNF to enter the brain [65,66,67,68], or that LITUS resulted in glial activation, and the increase in astroglia promoted increased endogenous GDNF. Importantly, LIFUS of either the STN or GPi restored motor behaviour and coordination damaged by MPTP or 6-OHDA, as assessed by the rotarod and pole tests, in both PD mice [40,47,49,50] and rat models [43]. This treatment effect continued to improve with increased LIFUS duration [40,47,49,50].

To evaluate the safety of LIFUS *in vivo*, all studies had employed the use of HE staining to assess the presence of haemorrhage or tissue damage and Nissl staining to visualise neurons in brain sections [40,46,48,49,50]. All studies reported the absence of haemorrhage, or cytotoxic damage with normal neuronal density throughout the brain [40,46,48,49,50].

## 4. Discussion

### 4.1. Summary of Findings

This work represents the first attempt to comprehensively review the available preclinical and clinical evidence pertaining to the function of FUS as a non-invasive and reversible treatment for PD worldwide, during established irreversible neuromodulatory stereotactive procedures, such as DBS, and ablative approaches, such as stereotactic radiosurgery and MRgFUS.

Our review demonstrates preclinical evidence of the safety and efficacy of FUS neuromodulation, but also highlights the paucity of *in vitro* evidence relating to its mechanisms of action.

### 4.2. Implications of Findings: Association and Causality

A causal relationship between FUS and neuromodulation of PD is tenable. Based on the Bradford Hill criteria [69], this may be supported by the strength and dose-dependent association reported in *in vitro* and *in vivo* preclinical studies [20,21,22,40,41,42,43,44,45,46,47,48,49,50,70,71,72,73,74]. The literature has consistently proven that FUS is capable of eliciting various behavioural responses in various PD models [40,43,47,49,50,72,73,74,75], inducing protein expression changes in GDNF [41,43], TH [40,41,43,46] and dopamine levels [46] in the SNpc, which are implicated in the disease pathology of PD. Indeed, the treatment effect continued to improve with increased LIFUS duration, even suggesting a dose–response relationship [40,47,49,50].

These phenomena are reinforced further by the biological plausibility of a mechanical bioeffect [76]. Although FUS is known to have a thermal effect, with the parameters used for neuromodulation [77] this is unlikely to play a major role. Generally, low-intensity protocols are employed (<20 W/cm2) for the purpose of neuromodulation, which is within Food and Drug Administration limits [70,73,78]. At such intensities, the accompanying temperature rise is deemed too miniscule (<0.1 °C) to potentiate neuromodulation [79], or even cause tangible thermal damage [70,72]. Studies have alternatively suggested that the most probable mechanism of FUS action is through acoustic radiation force (ARF) generation, which promotes neuronal membrane strain, consequently modulating its capacitance and its embedded mechanosensitive channel proteins [80,81,82]. Consistent with this notion, particular channels appear to be directly modulated by FUS include voltage-gated sodium, potassium and calcium channels [70,83], and channels of the Piezo family [84]. Additionally, there are speculations that LITUS resulted in glial activation, and the increase in astroglia promoted increased endogenous GDNF [65,66,67,68,85,86]. There is compelling evidence that the neuromodulatory effects elicited by LIFUS *in vivo* are mediated additionally by non-neuronal mechanisms such as glia-neuron interaction in the mammalian brain. Astrocytes, with their long peripheral processes, have intimate spatial relationships with presynaptic and postsynaptic synapses of neighbouring neurons (also known as the tripartite synapse) [87,88,89]. Dysregulation of the astrocyte–dopaminergic neuron interaction has a role in the pathophysiology of PD [90]. It is also well-established that the Ca^2+^-dependent release of glutamate from astrocytes stimulate neuronal NMDA receptors (NMDARs), modulating their synaptic activity [91,92,93]. Oh and colleagues reported that this FUS-induced neuromodulation is initiated by the opening of TRPA1 Ca^2+^ channels in astrocytes, which are pressure sensitive. This astrocytic influx of Ca^2+^ entry prompts the release of gliotransmitters such as glutamate through Best1 channels, which eventually activates the NMDAR of neurons in the vicinity to elicit action potential firing [91]. Interestingly, Blackmore et al. corroborated these findings in a recent study that showed FUS restored long-term potentiation and memory in senescent mice. Specifically, they demonstrated that FUS significantly raised levels of TRPA1 levels in synaptosomal hippocampal fractions compared with sham treatments [94]. This increase, reflected by the changes observed for NMDAR subunits in these fractions, suggests that ultrasound-mediated astrocytic glutamate release is a likely mechanism by which FUS led to the observed improvements in senescent mice, which may also explain the improvements in locomotor behaviour as seen in PD models [94].

### 4.3. Implication on the Direction of Future Research

FUS holds promise as a powerful neuromodulatory tool for PD and is hence attractive, and studies have investigated this in a multitude of ways. Consistent reporting of the neuromodulatory effects of FUS can be facilitated by an agreed minimum set of indicators to be reported, for example, changes in the expression of neuroprotective proteins for *in vitro* studies. Consistent reporting of these outcomes across future studies will enhance the validity of evidence syntheses [95]. By virtue of the fact that preclinical studies such as those we have highlighted rely on models of PD pathology that might not completely reflect disease mechanisms in humans, it is particularly important that future studies assessing the neuromodulatory effects of FUS for PD meet criteria spanning across the cellular, molecular and behavioural levels. This will help build confidence in the novel intervention being of relevance to the human disease state. Additionally, preclinical animal models typically include a behavioural component to assess the motor deficits characteristic of PD. These usually include locomotor deficits evident in various motor behaviour and coordination assays. There is a general consensus that the animal models offer compelling similarity in functional deficits to those found in humans [96]. Therefore, an important outcome measure for any future intervention studies would likely include demonstration of improvements in these key movement metrics. Table 2 outlines a proposed checklist for the outcome measures to directly assess the neuromodulatory effects of FUS for PD as a starting point. This checklist is intentionally generic, representing a minimum set of critically important outcomes to report in all studies evaluating the introduction and evaluation of FUS neuromodulation relevant to PD and should not restrict investigators in their reporting of additional relevant outcomes. In future, this could be further refined by a Delphi consensus of various stakeholders—scientists, electrophysiologists, neurologists and neurosurgeons.

This review additionally identified a current lack of *in vitro* studies that investigate the mechanistic changes induced by FUS. A significant caveat is that many of the *in vivo* models that assess the behavioural effects of FUS are confounded by the use of anaesthesia in their experimental design, which is unavoidable due to ethical reasons [45,46,97]. Kawaguchi et al. demonstrated that the typically used isoflurane dampens the motor-evoked potentials induced [98]. Of greater significance, several potential mechanisms of how FUS modulates membrane excitability coincide with those of the anaesthetic drugs [80]. As these studies employ the use of anaesthesia, the authors cannot exclude the possibility that the effects of FUS are altered under these conditions due to residual confounders [99]. Therefore, more *in vitro* studies such as those identified in this scoping review are recommended for more precise characterisation of the cellular mechanisms underpinning FUS. However, current *in vitro* studies, although captivating, are limited by the use of the PC12 cell culture to model PD. This is a pheochromocytoma cell line and does not exhibit the electrophysiological properties of post-mitotic midbrain dopaminergic neurons (mDANs). One way to circumvent this issue would be through the use of human-induced pluripotent stem cell line-derived mDANs [100], which enables a more accurate recapitulation of sporadic PD pathogenesis *in vitro* [101].

Chronic stimulation of deep brain structures using low-intensity FUS as a direct replacement for traditional electrode-based DBS removes the invasive procedure required for DBS but would impose the wearing of some form of targeted transducer device. However, given that certain FUS parameters seem to have effects lasting beyond the duration of stimulation, in both *in vitro* [102], and in *in vivo* PD models [49], we speculate on the possibility of finding a regular treatment protocol allowing for FUS to be given to the patient, which would induce a long-term therapeutic effect.

It is important to note that whilst the studies we identify specified no damaging effects of FUS, there remains the possibility, as with any neurostimulation tool, of functional adverse events. Whilst more work is therefore required to definitively establish this, it is nonetheless encouraging that, in a recent review of a number of human FUS studies, no evidence of lasting functional side effects or major adverse events was reported [103].

### 4.4. Strengths and Limitations

The findings are derived from a thorough search of three electronic databases. By including results from all study designs (preclinical and clinical), a comprehensive review of research evidence was achieved.

Only 11 studies qualified for inclusion in this review. Conference abstracts and other grey literature were excluded which may entrench publication bias or the ‘file-drawer problem’ [104]. However, during the screening process, conference abstracts rarely evinced sufficient details that would have been beneficial to this review. Nevertheless, we were still able to collate results in a structured manner and draw conclusions that represent a starting point for more robust future studies. We identified specific gaps in the existing literature, which are outlined in the ‘Implications of Findings and Direction of Future Research’ Section above.

## 5. Conclusions

This scoping review provides a starting point to better understand the research surrounding FUS in PD around the world. Preclinical evidence indicates that FUS is safe and has beneficial neuromodulatory effects on motor behaviour in PD. However, there is a current lack of mechanistic understanding for these beneficial effects of FUS in PD, either *in vivo* or *in vitro*; hence, there is need for further studies with more appropriate cellular models and clear reporting of our suggested outcome measures. FUS seems to have a role in influencing the disease process of PD, and therefore holds great promise as an attractive and powerful neuromodulatory tool for PD.

## Figures and Tables

**Table 1:** Characteristics and summary of findings from the included preclinical studies.

Authors and Year	Country	Study Design	Sample Size, Types of Subjects/Participants	Intervention (Control)	Outcome Measures/Indicators	Main Findings Relating to FUS in PD
Chen X et al., 2021	China	*In vivo and in vitro*	N = 5 per group. MPTP-induced C57BL/6 PD mice models. MPP+-induced N2a cells	LIFUS *In vivo*: 10 min of ultrasonic irradiation (every 24 h, 5 times) Frequency: 1 MHz Pulse repetition frequency: 1 kHz Duty cycle: 20%, for 10 min. Intensity: Grade 2 (123 ± 2.781± to 110.667 ± 3.138 mW/cm^2^.) *In vitro*: Frequency: 1 MHz Pulse repetition frequency: 1 kHz Duty cycle: 20%, for 10 min Intensity: Grade 1 (40.5 ± 1.857 ± to 40.3 ± 0.919 mW/cm^2^.)	*In vivo* measures: Locomotor behaviour Proportion of TH+ neurons in the SN_pc_ Neuronal activity Suppression of MPTP-induced cell apoptosis Morphological and pathological changes on brain sections *In vitro* measures: Suppression of MPP+-induced ROS generation Improvement in cell viability/reduction in apoptosis	Efficacy: In PD mice, LIFUS improved locomotor function In PD mice, LIFUS attenuated the central neurotoxicity of MPTP, reduced the loss of TH+ neurons and decreased the apoptosis in the section of SN_pc_ In N2a cells, low-intensity ultrasound protected against MPP+-induced neurotoxicity and mitochondrial membrane potential damage Safety: LIFUS did not cause any cytotoxicity and tissue damage as demonstrated by HE and Nissl staining
Dong Y et al., 2021	China	*In vivo*	N = 20. 6-OHDA-induced Sprague-Dawley hemi-PD rat models	LIFUS 10 min of ultrasonic irradiation (total of 200 trials) Frequency: 500 kHz Pulse repetition frequency: 1 kHz ISPPA: 2.6 W/cm^2^	FA and T2* values via MRI scanning Proportion of TH+ neurons in the SN_pc_ Proportion of GNDF+ neurons in the SN_pc_ Iron content in the SN_pc_	In hemi-PD rats, LIFUS had neuroprotective effects and reduced the damage of 6-OHDA-induced neurotoxicity LIFUS increased the proportion of TH+- and GDNF+-stained cells in the SN_pc_ In the 5th and 6th weeks post stimulation, LIFUS reduced FA values and increased T2* values
Karmacharya MB et al., 2017	Korea	*In vitro*	MPP^+^-induced PC12 cell PD models	Low-intensity ultrasound 10 min of ultrasonic irradiation (every 24 h) Frequency: 1 MHz Intensity: 30, 50, or 100 mW/cm^2^	Suppression of MPP^+^-induced α-Synuclein Phosphorylation and Aggregation Suppression of MPP^+^-induced ROS generation Attenuation of MPP^+^-induced suppression of mitochondrial complex I activity Suppression of MPP^+^-induced expression of CK2 Improvement in cell viability/reduction in apoptosis	In MPP^+^ induced PC12 cells, low intensity ultrasound attenuated mitochondrial ROS production and improved mitochondrial complex I activity Low intensity ultrasound decreased α-synuclein aggregation, levels of phosphorylated α-synuclein and CK2 expression Low intensity ultrasound improved cell viability assessed by MTT and TUNEL assay, after being treated with MPP^+^
Sung CY. et al., 2021	Taiwan	*In vivo*	N = 20. 6-OHDA-induced Sprague–Dawley PD rat models	LIFUS 5 min of ultrasonic irradiation Frequency: 1 MHz Pulse repetition frequency: 1 Hz Duty cycle: 5% Burst length: 5 ms ISPTA: 528 mW/cm^2^	Locomotor behaviour Proportion of TH+ neurons in the SN_pc_ Proportion of GNDF+ neurons in the SN_pc_ Proportion of BNDF+ neurons in the SN_pc_ Reduction of neuroinflammation	In PD rats, LIFUS improved locomotor function In PD rats, LIFUS increased the proportion of TH+ neurons in the striatum and SNPC In PD rats, LIFUS increased the levels of GDNF in the SN_pc_ but not BDNF levels. LIFUS attenuated LCN2-induced neuroinflammation
Tarnaud T et al., 2019	Belgium	Computational modelling study	A computational model for ultrasonic stimulation of the STN is created by combining the Otsuka model with the bilayer sonophore model	LIFUS Variable parameters explored	Parameter optimisation	In the STN, low intensities result in repetitive firing, while higher intensities result in silences Pulsed ultrasonic stimulation results in a shorter saturation latency and can modulate spiking rates
Wang Z et al., 2020	China	*In vivo*	N = 11. MPTP-induced C57BL/6 PD mice models	LIFUS 5 min of ultrasonic irradiation Frequency: 500 kHz Pulse repetition frequency: 1 kHz Duty cycle: 5% for 50 ms Intensity: Grade 2 (123 ± 2.781± to 110.667 ± 3.138 mW/cm^2^ ISPPA: 5.1 W/cm^2^ ISPTA: 0.255 W/cm^2^	Local field potentials in the motor cortex (M1)	In PD mice, LIFUS can influence important biomarkers of PD in M1. In the M1, LIFUS reduced the mean power intensity in the beta band In the M1, LIFUS reduced the PAC strength of both beta/high gamma and beta/ripple bands
Xu T et al., 2020	China	*In vivo and in vitro*	N = 12 per group. MPTP-induced C57BL/6 PD mice models and PC12 cells	LIFUS 10, 20, 30, 40, 50 and 60 s of ultrasonic irradiation Frequency: 1 MHz Pulse repetition frequency: 1 Hz Duty cycle: 5% Burst length: 5 ms Intensity: 30, 50, or 100 mW/cm^2^	*In vivo* measures: Locomotor behavior Locomotor behaviour Dopamine content in the SN_pc_ Proportion of TH+ neurons in the SN_pc_ Membrane permeability Morphological and pathological changes on brain sections *In vitro* measures: Dopamine release from PC12 cells	Efficacy In PC12 cells, low-intensity ultrasound enhanced DA release In PD mice, 10-day LIFUS enhanced DA content in the striatum In PD mice, LIFUS restored locomotor activity and enhanced the number of TH+ neurons in the SN_pc_ Safety LIFUS did not cause any cytotoxicity and tissue damage as demonstrated by HE and Nissl staining
Yuan Y et al. 2020	China	*In vivo*	N = 8 per group. MPTP-induced C57BL/6 PD mice models	LIFUS 5 min of ultrasonic irradiation Frequency: 500 kHz Pulse repetition frequency: 1 kHz Duty cycle: 5% for 50 ms Intensity: Grade 2 (123 ± 2.781± to 110.667 ± 3.138 mW/cm^2^ ISPPA: 5.1 W/cm^2^ ISPTA: 0.255 W/cm^2^	Locomotor behaviour	In PD mice, LIFUS improved the locomotor behaviour The treatment effect improved with increased LIFUS duration
Zhao L et al., 2017	China	*In vitro*	PC12 cells exposed to MPP^+^-induced neurotoxicity	Low-intensity ultrasound 10 min of ultrasonic irradiation Frequency: 1 MHz Pulse repetition frequency: 100 Hz Duty cycle: 20% for 10 min ISPTA: 50 mW/cm^2^	Suppression of MPP^+^-induced ROS generation Attenuation of MPP^+^-induced suppression of mitochondrial complex I activity Improvement in cell viability/reduction in apoptosis	In PC12 cells, low-intensity ultrasound inhibited MPP^+^-induced neurotoxicity and mitochondrial dysfunction Low-intensity ultrasound decreased MPP^+^-induced oxidative stress by modulating antioxidant proteins, including thioredoxin-1 and haem oxygenase-1, and prevented neurocytotoxicity via the phosphoinositide 3-kinase (PI3K)-Akt and ERK1/2 pathways. This neuroprotective effect was attributed to the activation of K2P channels and stretch-activated ion channels
Zhou H et al., 2019a	China	*In vivo*	N = 8. MPTP induced C57BL/6 PD mice models	LIFUS 30 min of ultrasonic irradiation daily Frequency: 3.8 MHz Pulse repetition frequency: 1 kHz Duty cycle: 50% for 10 min ISPTA: 430 mW/cm^2^	Locomotor behaviour Proportion of TH+ neurons in the SN_pc_ Neuronal activity Suppression of MPTP-induced cell apoptosis/reduction of antioxidant enzyme activity Morphological and pathological changes on brain sections	Efficacy In PD mice, LIFUS of the STN or GP_i_ improved motor behaviour LIFUS improved neuronal activity LIFUS stimulation of either STN or GP_i_ protected against MPTP-induced neurotoxicity in dopaminergic neurons by downregulating Bax, upregulating Bcl-2 and blocking cytochrome c release from mitochondria and reducing cleaved-caspase 3 activity in the SN_pc_ Safety LIFUS did not cause any cytotoxicity and tissue damage as demonstrated by HE and Nissl staining
Zhou H et al., 2019b	China	*In vivo*	N = 8. MPTP-induced C57BL/6 PD mice models	LIFUS 40 min of ultrasonic irradiation daily Frequency: 800 kHz Pulse repetition frequency: 100 Hz Duty cycle: 10% ISPPA: 760 mW/cm^2^	Locomotor behaviour Suppression of MPTP-induced cell apoptosis/reduction of antioxidant enzyme activity Neuronal activity Morphological and pathological changes on brain sections	Efficacy In PD mice, LIFUS improved locomotor activity LIFUS increased striatal total superoxide dismutase and glutathione peroxidase, important for the protection against MPTP-induced toxicity Safety LIFUS did not cause any cytotoxicity and tissue damage as demonstrated by HE and Nissl staining

6-OHDA = 6-hydroxydopamine; BDNF = Brain-derived neurotrophic factor; DBS = Deep brain stimulation; FA = Fractional anisotropy; GDNF = Glial cell line-derived neurotrophic factor; GPi = Globus pallidus internus; HE = Haematoxylin and eosin; ISPPA = Spatial peak and pulse-average intensity; ISPTA = Spatial peak and temporal average intensity; LDH = Lactate dehydrogenase; LFP = Local field potential; LIFUS = Low-intensity focused ultrasound; MPP^+^ = 1-methyl-4-phenylpyridinium; MPTP = 1-methyl-4-phenyl-1,2,3,6-tetrahydropyridine; MTT = 3-(4,5-dimethylthiazol-2-yl)-2,5-diphenyltetrazolium bromide; PD = Parkinson’s disease; ROS = Reactive oxygen species; SNpc = Substantia nigra pars compacta; STN = Subthalamic nucleus; TH = Tyrosine hydroxylase; TUNEL = Terminal deoxynucleotidyl transferase dUTP nick end labelling.

**Table 2:** A proposed checklist for the outcome measures to directly assess the neuromodulatory effects of FUS for PD.

Outcome Measures/Indicators
**Preclinical—*in vitro***
Changes in expression at the gene, RNA and protein level α-Synuclein phosphorylation and aggregation TH expression Dopamine expression GDNF expression Neuroinflammation
Changes in mitochondrial integrity ROS generation Mitochondrial complex I activity Membrane permeability Cell viability/reduction in apoptosis
**Preclinical—*in vivo***
Changes in expression at the gene, RNA and protein level TH expression in the SN_pc_ and striatum Dopamine expression in the SN_pc_ and striatum GDNF expression in the SN_pc_ and striatum
Electrophysiological and synaptic properties Local field potentials FA and T2* values via MRI scanning
Behavioral outcomes Rotarod test Vertical pole tests Open field test Forced swimming test
**Clinical**
Functional outcomes Procedure-related complications/adverse events Change in MDS-UPDRS part III motor score Change in UDysRS score Clinical improvement according to the patients’ global impression of change Neuropsychological effects assessed by quality-of-life questionnaire

FA = Fractional anisotropy; GDNF = Glial cell line-derived neurotrophic factor; MDS-UPDRS = Movement Disorder Society version of the United Parkinson’s Disease Rating Scale; MRI = Magnetic resonance imaging; PD = Parkinson’s disease; RNA = Ribonucleic acid; ROS = Reactive oxygen species; SN_pc_ = Substantia nigra pars compacta; STN = Subthalamic nucleus; TH = Tyrosine hydroxylase; UDysRS = Unified dyskinesia rating scale; UPDRS = Unified Parkinson’s disease rating scale; US = United States.

## Data Availability

Please find available data in the Supplementary Materials.

## Supplementary Materials

The following supporting information can be downloaded at: https://www.mdpi.com/article/10.3390/brainsci12020289/s1, Table S1: Search strategies across the four electronic databases as of 13 January 2022, Table S2: Inclusion and exclusion criteria used to assess eligibility of studies.
