# Supplementary material for: Focused Ultrasound Stimulation as a Neuromodulatory Tool for Parkinson’s Disease: A Scoping Review
Source: Brain Sci. 2022 Feb 19;12(2):289. doi: 10.3390/brainsci12020289 (PMC8869888; doi:10.3390/brainsci12020289)
Supplement: Supplementary file 1 [file brainsci-12-00289-s001.zip › brainsci-1575840-supplementary.pdf]

Table S1: Search strategies across the four electronic databases as of 13 January 2022

**OVID Medline 78**	
No.	Search term
**PD concept**	
1	exp Parkinson Disease/ or Parkinson disease.tw. or PD.tw.
**FUS neuromodulation concept**	
2	exp Ultrasonic Waves/ or exp Ultrasonic Therapy/ or ultrasound.tw. or focused ultrasound.tw. or LILFU.tw. or low intensity, low frequency ultrasound.tw.
3	(stimulation or neuromodulation).tw.
4	2 and 3
**Combined concept**	
5	1 and 4
**OVID Embase 264**	
No.	Search term

**PD concept**	
1	exp Parkinson Disease/ or Parkinson disease.tw. or PD.tw.
**FUS neuromodulation concept**	
2	exp focused ultrasound surgery/ or exp focused ultrasound therapy/ or exp low intensity focused ultrasound/ or exp low intensity pulsed ultrasound/ or exp low intensity ultrasound/ or exp MR-guided focused ultrasound/ or exp pulsed ultrasound/ or exp transcranial magnetic resonance guided focused ultrasound/ or exp ultrasound/ or exp<br>or ultrasound.tw. or focused ultrasound.tw. or LILFU.tw. or low intensity, low frequency ultrasound.tw.
3	exp neuromodulation/ or (stimulation or neuromodulation).tw.
4	2 and 3
**Combined concepts**	
5	1 and 4
**Web of Science 25**	

1	Parkinson Disease (All Fields) and focused ultrasound (All Fields) and neuromodulation (All Fields)
**Cochrane Central Register of Controlled Trials 6**	
No.	Search term
**PD concept**	
1	MeSH descriptor: [Parkinson Disease] explode all trees
2	(Parkinson disease):ti,ab,kw
3	#1 OR #2
**FUS neuromodulation concept**	
4	MeSH descriptor: [Ultrasonic Therapy] explode all trees
5	(low intensity focused ultrasound):ti,ab,kw
6	(focused ultrasound):ti,ab,kw
7	(LILFU):ti,ab,kw
8	#4 or #5 or #6 or #7
9	(neuromodulation):ti,ab,kw
10	(stimulation):ti,ab,kw

11	#9 or #10
12	#8 and #11
**Combined concepts**	
13	#3 and #12

Table S2: Inclusion and exclusion criteria used to assess eligibility of studies.

Inclusion criteria	Exclusion criteria
• Any published primary studies (preclinical or clinical using quantitative, qualitative or mixed methodology), reporting original data<br>• Studies on FUS in PD	• Non-primary studies: narrative reviews, systematic reviews and meta-analysis, editorials, commentaries, opinion papers, letters, education papers, conference abstracts, protocols, reports, theses or book chapters<br>• Studies not reporting data on FUS in PD at all<br>• Studies reporting data on FUS but not specific to PD<br>• Overlapping data

\
